# Effects of Diet Based on IgG Elimination Combined with Probiotics on Migraine Plus Irritable Bowel Syndrome

**DOI:** 10.1155/2019/7890461

**Published:** 2019-08-21

**Authors:** Yangzhi Xie, Guijuan Zhou, Yan Xu, Bing He, Yilin Wang, Rundong Ma, Yunqian Chang, Duanqun He, Chenlin Xu, Zijian Xiao

**Affiliations:** ^1^The First Affiliated Hospital of University of South China, University of South China, Hengyang, Hunan, China; ^2^Leiyang People's Hospital, Leiyang, Hengyang, Hunan, China

## Abstract

Several research studies have revealed that migraine has a solid link with gastrointestinal diseases especially irritable bowel syndrome (IBS). This study was carried out to investigate therapeutic potential of diet based on IgG elimination combined with probiotics on migraine plus irritable bowel syndrome. A total of 60 patients diagnosed with migraine plus IBS were recruited for the study. IgG antibodies against 266 food varieties were detected by ELISA. Then, the subjects were randomized into three groups for treatment of IgG elimination diet or probiotics or diet combined with probiotics. Migraine symptom, gut function score, medication use, and serum serotonin level were measured at baseline, 7 weeks, and 14 weeks. Improvement of migraine and gut symptom was achieved at a certain time point. Reduced use of over-the-counter- (OTC−) analgesics was seen in all groups. However, use of triptans did not show significant difference. An increased serum serotonin level was seen in subjects treated with elimination diet and elimination diet combined with probiotics. IgG elimination diet combined with probiotics may be beneficial to migraine plus IBS. It may provide new insight by understanding the intricate relationship between migraine and gastrointestinal diseases.

## 1. Introduction

Migraine is described as a debilitated headache with a prevalence of 13–33% over a lifetime. Patients may suffer severely from the symptoms as well as a high economic burden [1]. However, the underlying mechanisms are still not fully understood. There is growing evidence indicating that central nervous system (CNS) manifestations may appear after the gastrointestinal dysfunction [2]. The interactive relationship between the intestine and the brain is termed as the “gut-brain axis” [3]. Gratifying achievements have been made in delineating the bidirectional relationship between the CNS and the intestinal tract. Emerging evidence suggests that migraine patients tend to get gastrointestinal diseases and patients with gastrointestinal (GI) diseases are more liable to catch migraine, as compared to healthy controls [4–6]. Among these patients, migraine concomitant IBS is most commonly seen [7–9]. Growing evidence indicates that the intestinal microbiota and its metabolites may manage GI functions by affecting intestinal sensitivity and motility, intestinal permeability, and mucosal immune function [10, 11]. Undigested food particles and bacterial metabolite may enter the bloodstream and affect intestinal function [12], the leaky gut hypothesis suggests that intestinal disorders may prompt increased intestinal permeability, and then bacterial by-products such as lipopolysaccharides may flow into the bloodstream and ultimately cause a response provoking migraine [13, 14]. Moreover, intestinal microbiotas have been found to have a solid impact on neurotransmitter levels, especially serotonin (5-HT) which plays a significant role in migraine [15, 16]. Thus, amending function of the intestine may ameliorate intensity and duration time of migraine attacks. Probiotics, as living microorganisms, have been verified to stabilize the intestinal epithelial barrier in multiple ways [17]. Reduced pathogenic bacteria have been found when administered in probiotic bacterial strains by secreting antimicrobial factors. Furthermore, increased mucus output of the goblet cells has been found and they are of great importance for the tight junctions between the intestinal epithelial cells [18]. Several researchers found that diet based on elimination of certain food could reduce the occurrence and severity of migraine attacks [19, 20]. Abundance of food-specific IgGs may indicate food hypersensitivity. Hence, consumption of IgG-free food could ameliorate clinical manifestation of migraine.

Herein, we explored effects of diet based on IgG elimination combined with probiotics on migraine plus IBS, adding to growing evidence that management of intestinal function may be beneficial for migraine patients.

## 2. Materials and Methods

### 2.1. Subjects and Ethics

This study was carried out at The First Affiliated Hospital of University of South China. Sixty patients were enrolled in the study from May 2017 through December 2018 in the internal medicine department. International Classification of Headache Disorders, 3rd edition (beta version) (ICHD-3-beta), was employed to diagnose migraine; all patients were accompanied with uncomplicated IBS (bowel habit subtypes) according to the Rome III criteria. Five subjects were excluded due to difficulty in keeping the diet.

For meeting the inclusion criteria, the patients should (I) be aged between 18 and 65 years, (II) be diagnosed with migraine for more than 6 months and have at least 4 headache days within the last month, (III) have discomfort in the gut for more than 12 weeks in the past year, and (IV) be treated with preventive medications or acute attack medications unchanged for more than 6 months. Patients who have a definite history of medication overuse, headache, menstrual or other associated headache disorder, and organic abdominal diseases were excluded from the experiment.

Informed consent was obtained from subjects, and all the procedures were approved by the Institutional Review Board of the University of South China.

### 2.2. IgG Antibody Detection against Food Antigens and Diet Preparation

IgG antibodies against 266 food antigens were measured by a commercially available enzyme-linked immunosorbent assay (ELISA) kit (ImuPro 300 test; Evomed/R-Biopharm AG, Darmstadt, Germany). Quantitative measurements were reported in mg/l. Values above 7.5 mg/l were considered as positive reaction to the corresponding food. These samples were graded according to their titres, “low” for titres between 7.5–12.5 mg/l; “moderate” for 12.51–20 mg/l; “high” for 20.1–50 mg/l; and “very high” for 50.1–200 mg/l. According to the IgG antibody results, the elimination diet was composed of IgG negative food and the normal diet was made up with IgG-negative and IgG-positive food. There was no difference in calorie contents between these two diets. Subjects were guided to follow the diet (IgG-negative or IgG-positive) arranged by dietician.

### 2.3. Experiment Procedures and Measurement

A double-blind, randomized, controlled cross-over clinical trial was performed, and participants were randomly assigned to three groups which include subjects with elimination diet, probiotics, or elimination diet combined with probiotics. The probiotics product contains the following bacterial strains (*Bifidobacterium infantis*, *Lactobacillus acidophilus*, *Enterococcus faecalis*, and *Bacillus cereus*), and the subjects consumed 1.5 grams three times a day for 14 weeks.

During the experiment period, the subjects were requested to fill out a headache questionnaire, the Migraine Disability Assessment Scale (MIDAS), to evaluate severity of the migraine. IB Severity Scale (IBSS) was applied to assess the therapeutic effects of the intestine. A spectrophotofluorimetric method was applied to measure the concentration of serotonin in plasma [21]. Each scale and concentration of 5-HT in serum were assessed every 7 weeks.

### 2.4. Statistical Analysis

All data are expressed as mean ± S.D. Experiments with three or more groups were compared by ANOVA, followed by the LSD test. *p* < 0.05 was taken significant.

## 3. Results

### 3.1. Result of IgG Antibody Tests

Of the total 1506 reactions, 660 (43.8%) were graded as “low,” 693 (46%) were “moderate,” 105 (7%) were “high,” and 48 (3.2%) were “very high”. Food types are listed in Table 1.

### 3.2. Migraine Symptoms

**Figure 1:**
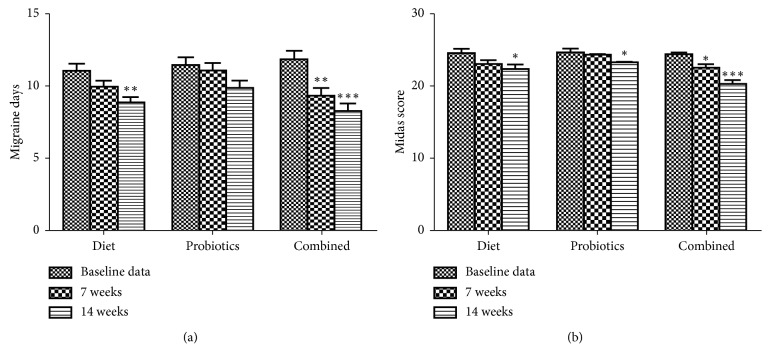
(a) Migraine days per 7 weeks at baseline and after 7 and 14 weeks of IgG elimination diet, oral intake of probiotics, or combined in migraine patients plus IBS. ^*∗∗*^*p* < 0.01 and ^*∗∗∗*^*p* < 0.001, compared to baseline data. (b) MIDAS score at baseline and after 7 and 14 weeks of IgG elimination diet, oral intake of probiotics, or combined in migraine patients plus IBS. ^*∗*^*p* < 0.05 and ^*∗∗∗*^*p* < 0.001, compared to baseline data.

As shown in Figure 1(a), migraine days of subjects with elimination diet in 14 weeks and elimination diet combined with probiotics in 7 and 14 weeks were significantly decreased. However, subjects with probiotics showed no difference. In 7 weeks, the mean MIDAS score decreased significantly only in subjects with elimination diet combined with probiotics. However, all groups exhibited an evident decrease in 14 weeks compared to baseline data (Figure 1(b)).

### 3.3. IBS Symptoms

**Figure 2:**
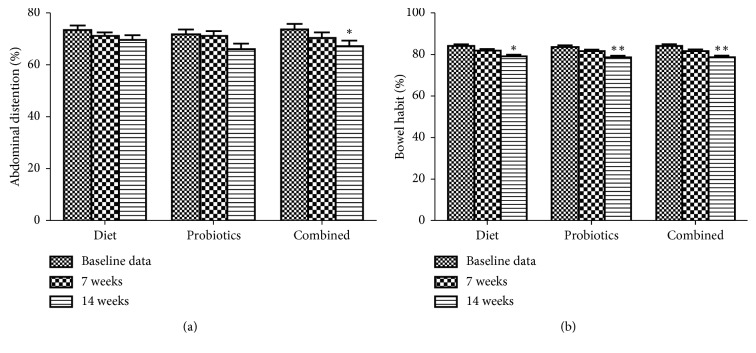
Abdominal distention and bowel habit score at baseline and after 7 and 14 weeks of IgG elimination diet, oral intake of probiotics, or combined in migraine patients plus IBS. ^*∗*^*p* < 0.05 and ^*∗∗*^*p* < 0.01, compared to baseline data.

As shown in Figure 2, in 14 weeks, a remarkable improvement was observed with all groups in bowel habit, compared to baseline data. No difference was found in 7 weeks. Only subjects with elimination diet combined with probiotics showed improvement in 14 weeks, referring to severity of abdominal distention.

### 3.4. Use of Medication

**Figure 3:**
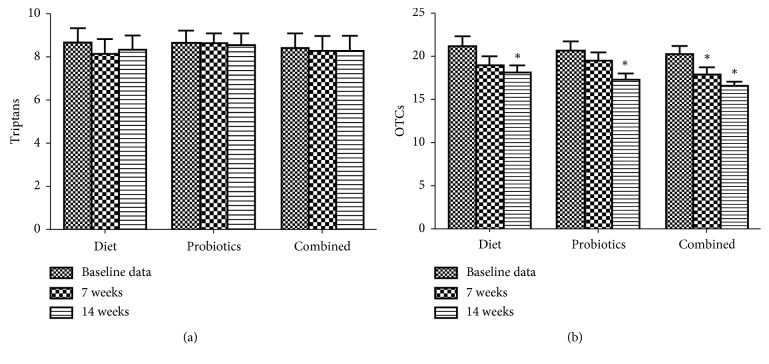
Medication use at baseline and after 7 and 14 weeks of IgG elimination diet, oral intake of probiotics, or combined in migraine patients plus IBS. ^*∗*^*p* < 0.05, compared to baseline data.

As shown in Figure 3, the use of triptans did not alter in all groups. The use of over-the-counter analgesics decreased in all groups in 14 weeks, only subjects with elimination diet combined with probiotics showed improvement in 7 weeks.

### 3.5. Concentration of 5-HT in Serum

**Figure 4:**
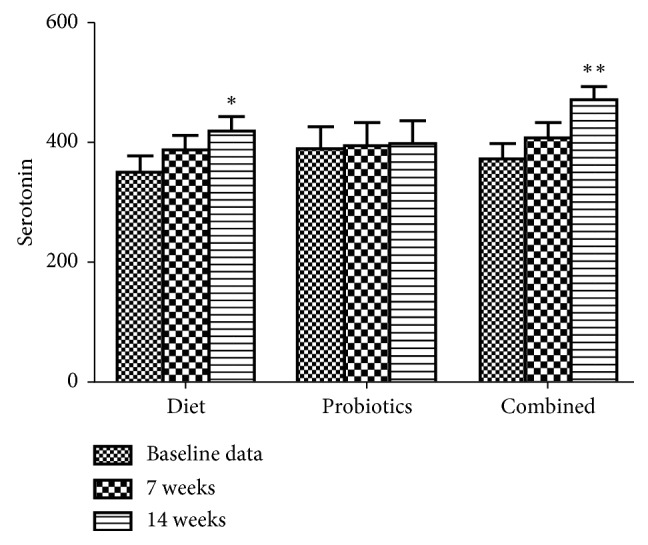
Serum level of serotonin at baseline and after 7 and 14 weeks of IgG elimination diet, oral intake of probiotics, or combined in migraine patients plus IBS. ^*∗*^*p* < 0.05 and ^*∗∗*^*p* < 0.01, compared to baseline data.

As shown in Figure 4, in 14 weeks, subjects with elimination diet or elimination diet combined with probiotics exhibited a significant increase in concentration of 5-HT in serum compared to baseline data. No difference was found in other groups and time points.

## 4. Discussion

To our knowledge, it is the first research to prove that IgG elimination diet combined with probiotic may be beneficial to migraine plus IBS. Meanwhile, compliance was high and relevant adverse reactions did not happen. According to our findings, IgG-mediated food allergies have been proved to play an important role in migraine attacks although the mechanism is not fully illuminated. There is emerging proof that inflammation acts as a crucial role in the pathogenesis of migraine [22, 23]. A specific marker is needed if we focus on inflammation response prompted by certain foods. All IgG subclasses except IgG4 cause inflammatory response after contact with specific antigen [24]. Thus, identifying IgG for variety of foods may be applicable to detect allergized food and give guidance for amendment of dietary habits so as to keep away from chronic inflammation and onset of migraine. IBS patients were proved to have a greater gut permeability defect than healthy controls. Thus, increased intake of dietary antigens to lamina propria occurred in individuals with IBS. Decreased lymphocyte proliferation and release of inflammatory cytokines were found when consuming customized elimination diet [25]. Several studies have indicated that probiotics have therapeutic efficacy in gastrointestinal diseases [26, 27]. The potential mechanism of probiotics in treating gut-associated diseases may strengthen intestinal barrier function in several ways. Meanwhile, it may impact pain pathways by influencing brain signaling [28]. Hence, probiotics may relieve migraine headache by amending the intestinal barrier function through the gut-brain axis. Serotonin is neurogenic and serves a pivotal role in cell differentiation, division, and migration [29]. The enteric nervous systems are made up of more than 100 million neurons, and they may communicate with the central nervous system bidirectionally and continuously through several mediators [30]. Of these mediators, serotonin is mostly researched. Several studies suggest that serotonin is an important link in the brain-gut axis. However, only 3% of the whole serotonin of human is located in the central nervous system. The rest is located in the intestine. Enteric bacteria have been elucidated to regulate production of serotonin. Therefore, amending the function of intestine may be a way to cure migraine patients. The migraine days significantly decreased in the diet group in 7 and 14 weeks and got better with time. Also, the diet group exhibited significant change in 14 weeks. Although decrease was seen in the probiotic group, it showed no statistical significance. This may be explained as probiotics may take effect slower than other groups. Improvement was seen in the combined group in 14 weeks, referring to abdominal distention; however, other groups did not show a significant change because the treatment was inadequate. Reduction in triptan use was not seen in all groups, and this may be explained as some subjects were hard to get rid of it due to drug dependence.

Our data are in consistence with other studies which report IgG elimination diet or probiotics are beneficial for migraine plus IBS. Compared to these studies, ours has many superiorities. To begin with, we found that subjects with IgG elimination diet combined with probiotics had effect quicker than other groups when considering migraine symptom (MIDAS score and migraine days). Secondly, although all the groups did not reduce the use of triptans, use of over-the-counter analgesic decreased dramatically in all groups in 14 weeks and only diet combined with probiotics showed effect at 7 weeks, and this indicated it may show effects quicker than other groups in nonacute attack. Moreover, improvement in bowel habit was seen in all groups in 14 weeks, but only subjects with diet combined with probiotics exhibited a relief in severity of abdominal distention. Thus, diet combined with probiotics may be an optimal selection for the management of intestinal function. Finally, titres of serotonin were upregulated in 14 weeks in subjects with diet and diet combined with probiotics, but the latter showed a greater magnitude. However, we must pay attention when translating these results into daily practice due to limited sample size. Further study is required to elucidate the underlying mechanism of IgG-positive, food-induced migraine.

## 5. Conclusions

In summation, we provide the first clinical evidence that IgG elimination diet combined with probiotics may be beneficial to migraine plus IBS. Future work should uncover the potential mechanism of how it affects pathophysiology of migraine.

## Figures and Tables

**Table 1:** Types of food from most to least frequent IgG positivity in patients.

Food types	Number of patients with positive test result (*n* = 60)
Spices	53
Seeds and nuts	50
Grain with gluten	48
Seafood	43
Food additives	26
Eggs	26
Cheese	24
Sugar products	24
Milk product	24
Grain without gluten	19
Vegetable	14
Coffee infusions	10
Salads	5
Yeast	5
Meat	5
Mushrooms	3

## Data Availability

The original data are available from the corresponding author upon request.
